# Association of plant-based food intake in daily diets and hypertension in older adults: a cohort study

**DOI:** 10.3389/fpubh.2025.1651399

**Published:** 2025-10-28

**Authors:** Xiang Wang, Ting Liu, Jianbang Shi, Wei Jie, Miao Dai

**Affiliations:** ^1^Department of Cardiology and Jiujiang City, Key Laboratory of Cell Therapy, Jiujiang People’s Hospital, Jiujiang, Jiangxi, China; ^2^Department of Cardiovascular and Neurology, Jiujiang Sixth People’s Hospital, Jiujiang, Jiangxi, China; ^3^Department of Geriatrics and Jiujiang City, Key Laboratory of Cell Therapy, Jiujiang NO.1 People’s Hospital, Jiujiang, Jiangxi, China; ^4^Chronic Disease Management Center, Jiujiang People’s Hospital, Jiujiang, Jiangxi, China

**Keywords:** plant-based diet, hypertension, older adults, cohort study, diet index

## Abstract

**Introduction:**

Hypertension is a major health concern among older adults, linked to high morbidity and mortality. While plant-based diets may offer health benefits, their association with hypertension in this population remains unclear. This study examines the relationship between plant-based food intake and hypertension incidence in older adults.

**Methods:**

We analyzed data from the Chinese Longitudinal Healthy Longevity Survey, including 3,991 hypertension-free participants aged ≥65 years at baseline (2008). Follow-up was conducted in 2011/2012. Plant-based diet intake was assessed using a plant-based diet index (PDI). Cox proportional hazard models estimated hazard ratios (HRs) and 95% confidence intervals (CIs) for hypertension risk.

**Results:**

During a mean follow-up of 3.0 years, 1,764 individuals (44.2%) developed hypertension. Stratified by median PDI, the high PDI group had a 16% lower risk of hypertension versus the control group. Compared to the first quartile of PDI, the highest quartile had a lower risk of hypertension (HR 0.79, 95% CI: 0.69–0.90). The third and second quartiles of PDI had HRs of 0.79 (0.69–0.91) and 0.86 (0.76–0.98), respectively. Subgroup analyses indicated that the relationship between PDI and hypertension risk was not influenced by sex, gender, marital status, living arrangement, economic status, or activities of daily living limitations.

**Discussion:**

Higher adherence to a plant-based diet was significantly associated with a reduced risk of hypertension in older adults, suggesting that dietary interventions emphasizing plant-based foods may help mitigate hypertension incidence in this population.

## Introduction

Hypertension is a leading risk factor for cardiovascular diseases and is highly prevalent among older adults, contributing significantly to global morbidity and mortality (1). With aging populations worldwide, identifying modifiable dietary factors to mitigate hypertension risk is a public health priority. Plant-based diets, characterized by higher consumption of fruits, vegetables, legumes, nuts, and whole grains, have gained attention for their potential cardioprotective effects. However, the specific association between plant-based food intake and hypertension incidence in older adults remains underexplored.

Previous studies have suggested that greater consumption of healthful plant foods and reduced intake of animal-source foods may contribute to hypertension prevention. For instance, a study found that a diet rich in healthful plant-based foods was associated with a lower risk of hypertension in middle-aged adults (2). Similarly, a cohort study demonstrated that vegetarians had a lower incidence of hypertension compared to nonvegetarians, potentially due to factors beyond abdominal obesity, inflammation, and insulin resistance (3). A meta-analysis further supported these findings, reporting that vegetarian diets were associated with lower blood pressure compared to omnivorous diets (4). However, these studies primarily included middle-aged or mixed-age populations, with limited representation of older adults. In contrast, a 2024 meta-analysis by Xia X et al., which included both observational studies and randomized controlled trials across diverse age groups (not exclusively older adults), found no significant association between vegetarian diets and systolic or diastolic blood pressure (5). This inconsistency underscores the need for targeted research in older populations, who may exhibit unique physiological responses to dietary patterns due to age-related changes in metabolism, nutrient absorption, and chronic disease profiles. Additionally, most existing studies have been conducted in Western countries, where dietary habits, such as higher consumption of processed foods and red meat, differ markedly from traditional Chinese diets, which emphasize grains, vegetables, and plant-based proteins.

Given the aging global population and the rising prevalence of hypertension, understanding the role of plant-based diets in hypertension prevention among older adults is crucial. This study aims to address this gap by examining the association between plant-based food intake and hypertension in a cohort of older adults in China, providing insights into culturally relevant dietary interventions for this vulnerable population.

## Methods

### Study design and participants

The Chinese Longitudinal Healthy Longevity Survey (CLHLS) is a prospective cohort study initiated in 1998 to investigate determinants of healthy aging and longevity among older adults in China. The study spans 22 out of 31 provinces, representing approximately 85% of the Chinese population. Follow-up surveys were conducted in 2000, 2002, 2005, 2008–2009, 2011–2012, 2014, and 2017–2018. To address attrition due to mortality and loss to follow-up, new participants were recruited at each follow-up, and surviving participants were re-interviewed. Data collection was carried out through structured questionnaires administered by trained interviewers at participants’ homes. Further details on the CLHLS design and methodology have been published elsewhere (6, 7).

**Figure 1:**
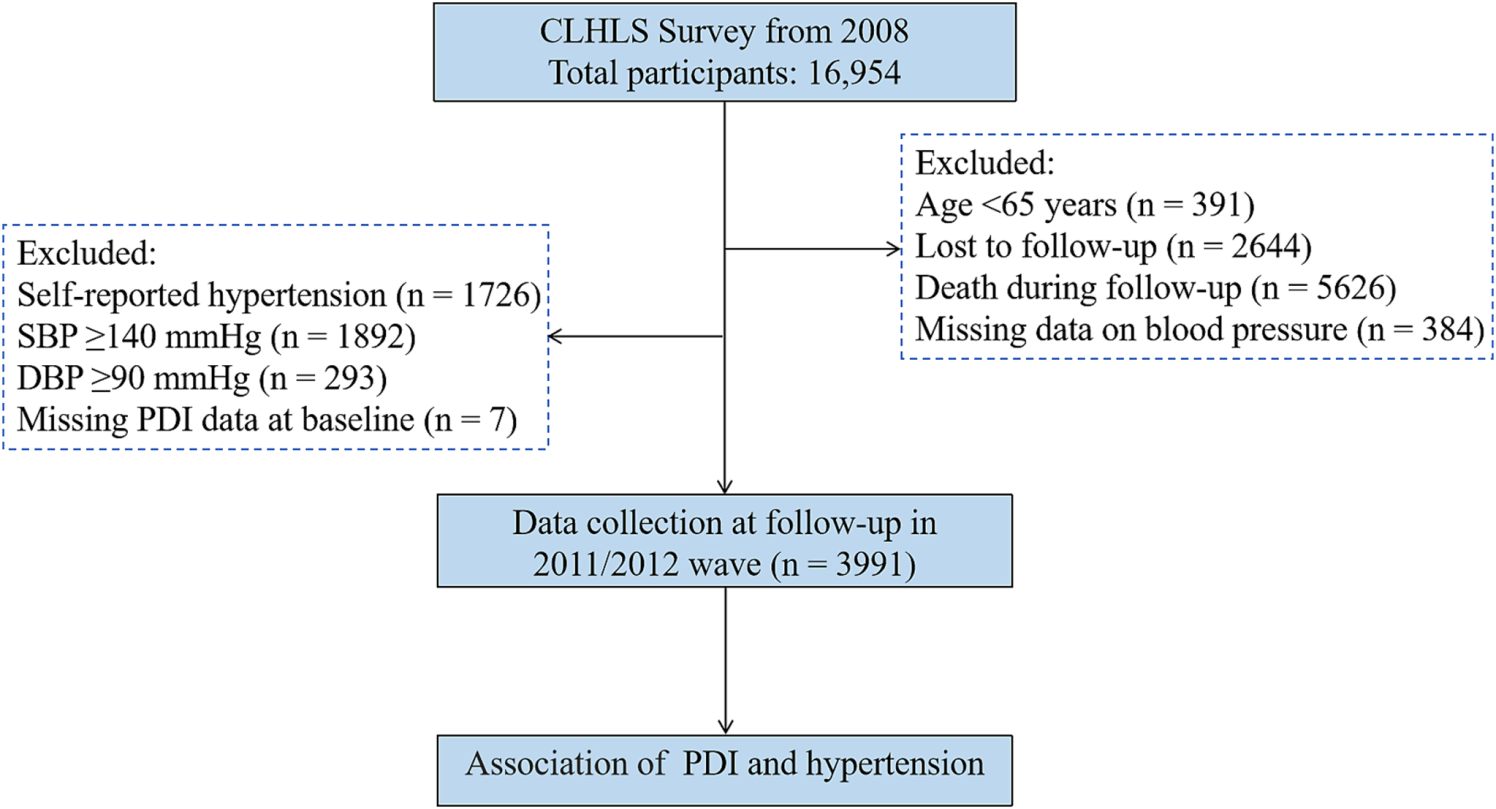
Flowchart of the included study population. CLHLS, Chinese Longitudinal Healthy Longevity Survey; SBP, systolic blood pressure; DBP, diastolic blood pressure; PDI, plant-based diet index.

For this study, data from the fifth wave of the CLHLS (2008) were utilized, as earlier waves lacked results from two blood pressure measurements, which are critical for defining hypertension. Follow-up data from 2011 and 2012 were also included. A total of 16,954 older adults were initially enrolled in the study. To examine the association between plant-based food intake and hypertension, participants were excluded based on the following criteria: age <65 years (*n* = 391), loss to follow-up (*n* = 2,644), death during follow-up (*n* = 5,626), missing blood pressure data (*n* = 384), physician-diagnosed hypertension at baseline (*n* = 1,726), systolic blood pressure (SBP) ≥ 140 mmHg and/or diastolic blood pressure (DBP) ≥ 90 mmHg at baseline (*n* = 2,185), and missing data on the plant-based diet index (PDI) (*n* = 7). After exclusions, 3,991 participants without hypertension at baseline were included in the final cohort. A flowchart detailing the inclusion and exclusion process is provided in Figure 1.

### Dietary pattern assessment

Dietary intake was assessed at baseline using a simplified food frequency questionnaire designed to capture consumption of foods commonly consumed in the Chinese diet. The questionnaire included two categories: plant-based foods (fresh fruits, fresh vegetables, vegetable oils, bean products, mushrooms, garlic, nuts, tea, sugar, and salt-preserved vegetables) and animal-based foods (animal fats, meat, fish, eggs, and dairy products). Participants reported the frequency of consumption for each food item at present.

The frequency of intake was categorized and scored as follows (Supplementary Table 1): for bean products, mushrooms, garlic, nuts, tea, sugar, salt-preserved vegetables, meat, fish, eggs, and dairy products, responses were classified into five categories: “almost every day,” “not every day, but at least once per week,” “not every week, but at least once per month,” “not every month, but occasionally,” and “rarely or never.” For fresh fruits and vegetables, responses were categorized into four groups: “almost daily,” “fairly often,” “occasionally,” and “rarely or never.” Plant-based foods were scored from 5 (highest frequency) to 1 (lowest), while animal-based foods were inversely scored (5 for lowest frequency). Vegetable oil received 5 points, and animal fat received 1 point. The PDI was calculated by summing scores across all food categories, with a theoretical range of 14–70 points. Higher PDI scores indicated greater adherence to a plant-based diet. Participants were categorized using PDI as a continuous variable, a binary variable (high level PDI vs. Low level PDI based on the median score), and quartiles (Q1–Q4) to assess associations with hypertension.

### Outcome assessment

Hypertension was the primary outcome, assessed through blood pressure measurements at baseline and follow-up. SBP and DBP were measured in mmHg using a mercury sphygmomanometer (upper arm type; Yuyue, Jiangsu, China). Participants rested for ≥5 min before measurement, with ≥1 min intervals between readings. The average of two consecutive measurements was used for analysis. Hypertension was defined as SBP ≥ 140 mmHg, DBP ≥ 90 mmHg, or a self-reported physician diagnosis. These criteria were considered complementary; a participant meeting any one criterion was classified as a hypertension case. This approach captures individuals with medicated and controlled hypertension (who may have normal measured BP) as well as those with undiagnosed hypertension (elevated measured BP without a self-reported diagnosis).

### Covariates assessment

Potential confounders were assessed by incorporating baseline sociodemographic characteristics, lifestyle factors, and health status, informed by existing literature. Sociodemographic covariates included age (continuous), sex (male or female), marital status (married or others), residence (rural, urban, or town), education (0 years, 1–6 years, ≥6 years), living with family members (yes or no), and economic status (independence or dependence). Lifestyle factors comprised smoking (never, current, or former), alcohol consumption (never, current, or former), regular exercise (never, current, or former), and sleep duration (<6 h, 6–9 h, or ≥9 h). Health status covariates included body mass index (BMI), categorized as underweight (<18.5 kg/m^2^), normal weight (18.5–24 kg/m^2^), overweight (24–28 kg/m^2^), or obese (≥28 kg/m^2^) (8), activity of daily living (ADL) limitation (yes or no), number of natural teeth (20+, 10–19, 1–9, 0), denture use (yes or no), and presence of chronic conditions such as heart disease, cerebrovascular disease, diabetes mellitus, respiratory disease, and cancer (all yes or no). ADL limitation was defined as experiencing difficulty or requiring assistance with at least one of six daily activities (bathing, dressing, indoor transferring, toileting, eating, and continence) due to health problems in the previous 6 months.

### Statistical analysis

Baseline characteristics were presented as means and standard deviations (SDs) for continuous variables and as percentages for categorical variables. Missing data, ranging from 0.10 to 0.45%, were addressed using multiple imputations based on 5 replications and the chained equation approach (Supplementary Table 2).

Cox proportional hazards regression models were employed to estimate hazard ratios (HRs) and 95% confidence intervals (CIs) for the association between the PDI and hypertension risk. PDI was analyzed as continuous, two-category (median as cut-off), and four-category (quartiles as cut-off) variables. The proportional hazards assumption was tested and satisfied. Initially, the unadjusted association between PDI and hypertension was examined. Adjustments were made in three sequential models: Model 1 adjusted for age and sex; Model 2 further adjusted for marital status, education, residence, living arrangement, economic status, smoking, drinking, regular exercise, body mass index, ADL limitation, sleep time, number of natural teeth, and denture use; and Model 3 additionally adjusted for heart disease, cerebrovascular disease, diabetes mellitus, respiratory disease, and cancer. Crude incidence rates of hypertension per 100 person-years across PDI categories were calculated. A restricted cubic spline analysis with four knots at the 5th, 35th, 65th, and 95th percentiles of PDI distribution was conducted to explore the dose–response relationship, using the 25th percentile of PDI (48 scores) as the reference. Interaction terms between potential covariates (age, sex, living arrangement, marital status, and ADL limitation) and PDI were tested, with subgroup analyses performed and *p*-values adjusted using Bonferroni corrections.

Sensitivity analyses were conducted to ensure robustness. First, complete-case analysis was performed to assess the impact of multiple imputation. Second, propensity score matching (PSM) was used to balance baseline characteristics between high-PDI and low-PDI groups, with standardized mean differences (SMDs) < 0.1 indicating balanced covariates. Lastly, participants with baseline chronic conditions (heart disease, diabetes mellitus, cerebrovascular disease, respiratory disease, or cancer) were excluded to minimize confounding.

All analyses were performed using R (version 4.2.2), with statistical significance set at a two-tailed *p*-value <0.05.

## Results

### Basic characteristics of participants

Among the 3,991 participants, 53.1% were female, with a mean (SD) age of 82.82 ± 11.09 years. Over a median follow-up period of 3.0 years (totaling 12,116.9 person-years), 1,764 incident hypertension cases (44.2%) were identified. Table 1 presents the baseline characteristics of participants stratified by their PDI levels. Participants with higher PDI scores were more likely to be younger, reside in urban areas, be married, live with family, engage in regular physical activity, attain higher education levels, and maintain economic independence compared to those with lower PDI scores. Additionally, individuals with higher PDI scores exhibited better oral health (e.g., more natural teeth and greater denture use), lower prevalence of underweight, fewer ADL limitations, and a reduced likelihood of respiratory diseases.

**Table 1:** Baseline characteristics of the participants.

Characteristics	Total (*n* = 3,991)	Plant-based diet index		*p* value
		Low level (*n* = 1,847)	High level (*n* = 2,144)	
Age (year), mean (SD)	82.82 (11.09)	83.94 (11.14)	81.86 (10.97)	<0.001
Female, no. (%)	2,118 (53.1)	985 (53.3)	1,133 (52.8)	0.784
Residence, no. (%)				<0.001
Urban area	639 (16.0)	221 (12.0)	418 (19.5)	
Town area	885 (22.2)	354 (19.2)	531 (24.8)	
Rural area	2,467 (61.8)	1,272 (68.9)	1,195 (55.7)	
Married, no. (%)	1,720 (43.1)	739 (40.0)	981 (45.8)	<0.001
Living arrangement, no. (%)				0.046
Living alone	674 (16.9)	336 (18.2)	338 (15.8)	
Living with family	3,317 (83.1)	1,511 (81.8)	1,806 (84.2)	
Smoking status, no. (%)				0.303
Never	2,566 (64.3)	1,209 (65.5)	1,357 (63.3)	
Current	852 (21.3)	387 (21.0)	465 (21.7)	
Former	573 (14.4)	251 (13.6)	322 (15.0)	
Drinking status, no. (%)				0.512
Never	2,640 (66.1)	1,239 (67.1)	1,401 (65.3)	
Current	848 (21.2)	382 (20.7)	466 (21.7)	
Former	503 (12.6)	226 (12.2)	277 (12.9)	
Regular exercise, no. (%)				<0.001
Never	2,272 (56.9)	1,130 (61.2)	1,142 (53.3)	
Current	1,301 (32.6)	519 (28.1)	782 (36.5)	
Former	418 (10.5)	198 (10.7)	220 (10.3)	
Education (year), no. (%)				<0.001
0	2,231 (55.9)	1,093 (59.2)	1,138 (53.1)	
1–6	1,305 (32.7)	572 (31.0)	733 (34.2)	
>6	455 (11.4)	182 (9.9)	273 (12.7)	
Economic independence, no. (%)	1,228 (30.8)	470 (25.4)	758 (35.4)	<0.001
Number of natural teeth, no. (%)				<0.001
<10	2,325 (58.3)	1,180 (63.9)	1,145 (53.4)	
10–20	764 (19.1)	305 (16.5)	459 (21.4)	
≥20	902 (22.6)	362 (19.6)	540 (25.2)	
Denture use, no. (%)	1,207 (30.2)	529 (28.6)	678 (31.6)	0.044
BMI (kg/m^2^), no. (%)				<0.001
Underweight (<18.5)	2,243 (56.2)	953 (51.6)	1,290 (60.2)	
Normal (18.5–24)	1,283 (32.1)	717 (38.8)	566 (26.4)	
Overweight (24–28)	375 (9.4)	141 (7.6)	234 (10.9)	
Obese (≥28)	90 (2.3)	36 (1.9)	54 (2.5)	
ADL limitation, no. (%)	357 (8.9)	190 (10.3)	167 (7.8)	0.007
Diabetes mellitus, no. (%)	67 (1.7)	26 (1.4)	41 (1.9)	0.265
Heart disease, no. (%)	215 (5.4)	102 (5.5)	113 (5.3)	0.779
Cerebrovascular disease, no. (%)	136 (3.4)	65 (3.5)	71 (3.3)	0.785
Respiratory disease, no. (%)	401 (10.0)	207 (11.2)	194 (9.0)	0.027
Cancer, no. (%)	10 (0.3)	4 (0.2)	6 (0.3)	0.935

BMI, body mass index; ADL, activity of daily living.

Values are presented as number (%) or mean ± SD. Differences in characteristics were compared using the *χ*^2^ test for categorical variables and t-test for continuous variables.

After PSM, 3,212 participants were included in the final analysis. All SMDs for covariates were below 0.1, indicating well-balanced groups with minimal confounding effects (Supplementary Figure 1). Post-PSM, baseline characteristics between low- and high-PDI groups were effectively balanced (Supplementary Table 3).

### Association of PDI with incident hypertension

After adjusting for potential confounding factors, participants with a high plant-based diet index (PDI) exhibited a significantly lower risk of hypertension compared to those with a low PDI (IR per 100 person-years: 14.2 vs. 15.0; HR = 0.84, 95% CI = 0.76–0.92) (Table 2).

**Table 2:** Hazard ratios for hypertension according to plant-based diet index (PDI) levels.

PDI category	Number of events/ total	Incidence rate ^a^	Unadjusted model	Model 1	Model 2	Model 3
			HR (95% CI)	HR (95% CI)	HR (95% CI)	HR (95% CI)
By median						
Low level (<48)	835/1,847	15.0	Reference	Reference	Reference	Reference
High level (≥48)	929/2,144	14.2	0.84 (0.77–0.93)	0.85 (0.77–0.93)	0.84 (0.76–0.92)	0.84 (0.76–0.92)
By Quartile						
Q1 (Lowest, ≤43)	473/1,029	15.2	Reference	Reference	Reference	Reference
Q2 (43–48)	467/1,057	14.6	0.89 (0.78–1.01)	0.89 (0.78–1.01)	0.86 (0.76–0.98)	0.86 (0.69–0.98)
Q3 (48–53)	439/1,008	14.3	0.81 (0.71–0.92)	0.81 (0.71–0.93)	0.79 (0.70–0.91)	0.79 (0.69–0.91)
Q4 (Highest, >53)	385/897	14.1	0.80 (0.70–0.92)	0.80 (0.70–0.92)	0.79 (0.69–0.91)	0.78 (0.69–0.90)
*p* for trend			<0.001	<0.001	<0.001	<0.001
Per 1-point increase			0.99 (0.98–0.99)	0.99 (0.98–1)	0.99 (0.98–0.99)	0.99 (0.98–0.99)

HR, hazard ratio; CI, confidence interval.

^a^Incidence rates per 100 person-years.

Model 1: adjusted for baseline age and sex.

Model 2: further adjusted for marital status, education, residence, living arrangement, economic status, smoking status, drinking status, regular exercise, body mass index, activity of daily living limitation, sleep time, the number of natural teeth, and denture use.

Model 3: further adjusted for heart disease, cerebrovascular disease, diabetes mellitus, respiratory disease, and cancer.

Compared to participants in the lowest PDI quartile (Q1), those in higher quartiles (Q4, Q3, Q2) demonstrated a 22.0, 21.0, and 14.0% reduction in hypertension risk, respectively (Table 2). A trend test, adjusted for potential confounders, confirmed a statistically significant linear association between PDI and hypertension risk (*p* < 0.001). For each 1-point increase in PDI, the risk of hypertension decreased by 1.0% (Table 2).

**Figure 2:**
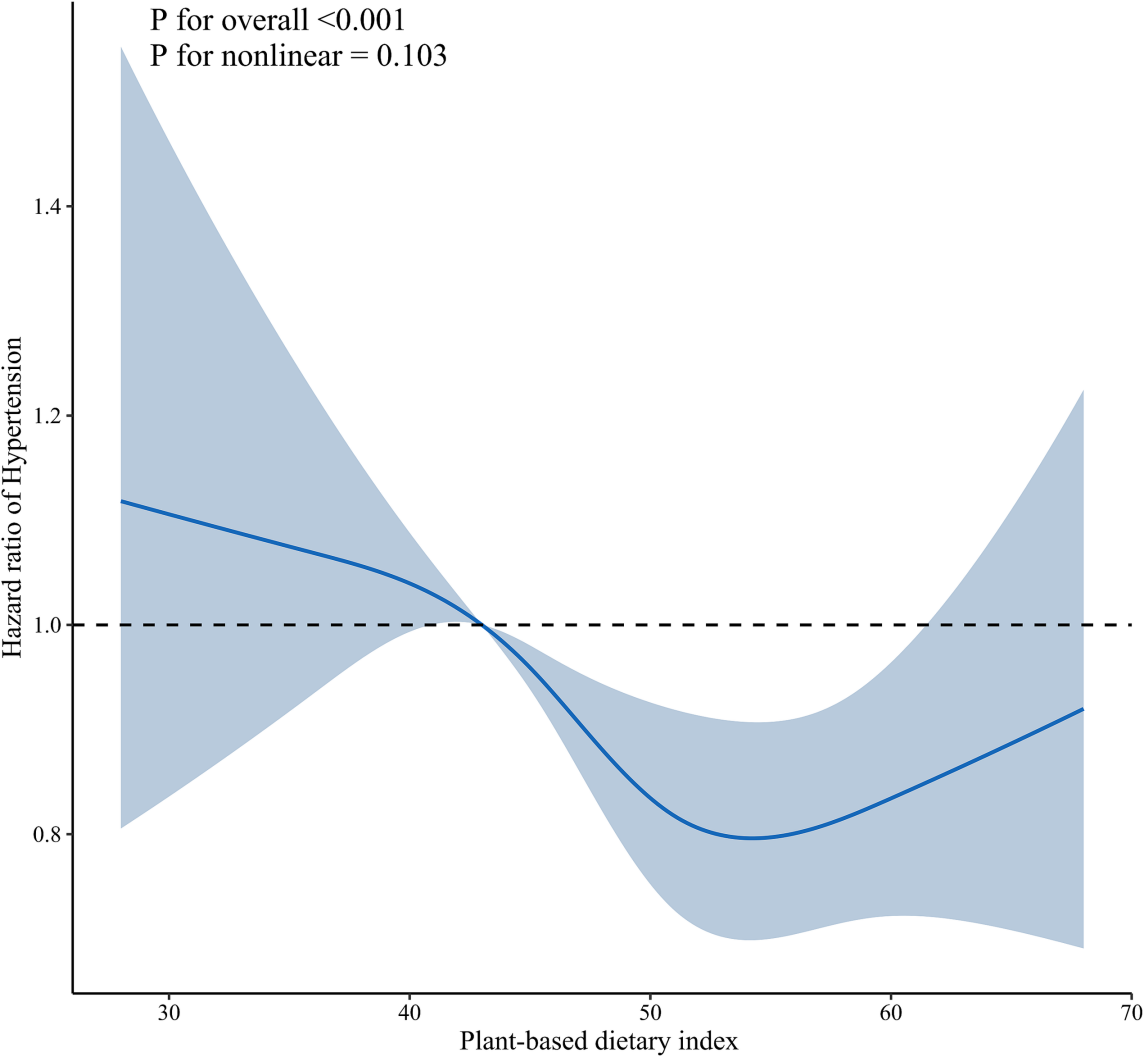
Dose–response association between plant-based diet index and hypertension. Solid blue lines are multivariable-adjusted hazard ratios, with shaded areas showing 95% confidence intervals derived from restricted cubic spline regressions with four knots. Multivariate models were adjusted for baseline age, sex, marital status, education, residence, living arrangement, economic status, smoking status, drinking status, regular exercise, body mass index, activity of daily living limitation, sleep time, the number of natural teeth, denture use, heart disease, cerebrovascular disease, diabetes mellitus, respiratory disease, and cancer.

Results from the restricted cubic spline analysis (Figure 2) revealed a non-linear, J-shaped relationship between continuous PDI scores and the risk of hypertension. The risk of hypertension decreased with increasing PDI scores up to a value of approximately 61.4. Within this range (PDI scores from ~43.0 to 61.4), higher adherence to a plant-based diet was associated with a progressively lower hazard of developing hypertension. The association was statistically significant, as indicated by a *p* for non-linearity of 0.034. However, beyond a score of 61.4, the risk of hypertension appeared to plateau and showed a non-significant tendency to increase, forming the upward curve of the “J.” This suggests that while a strong adherence to a plant-based diet is beneficial, there may be a point of diminishing returns or other factors at play at the very highest levels of the index.

### Subgroup analyses

**Figure 3:**
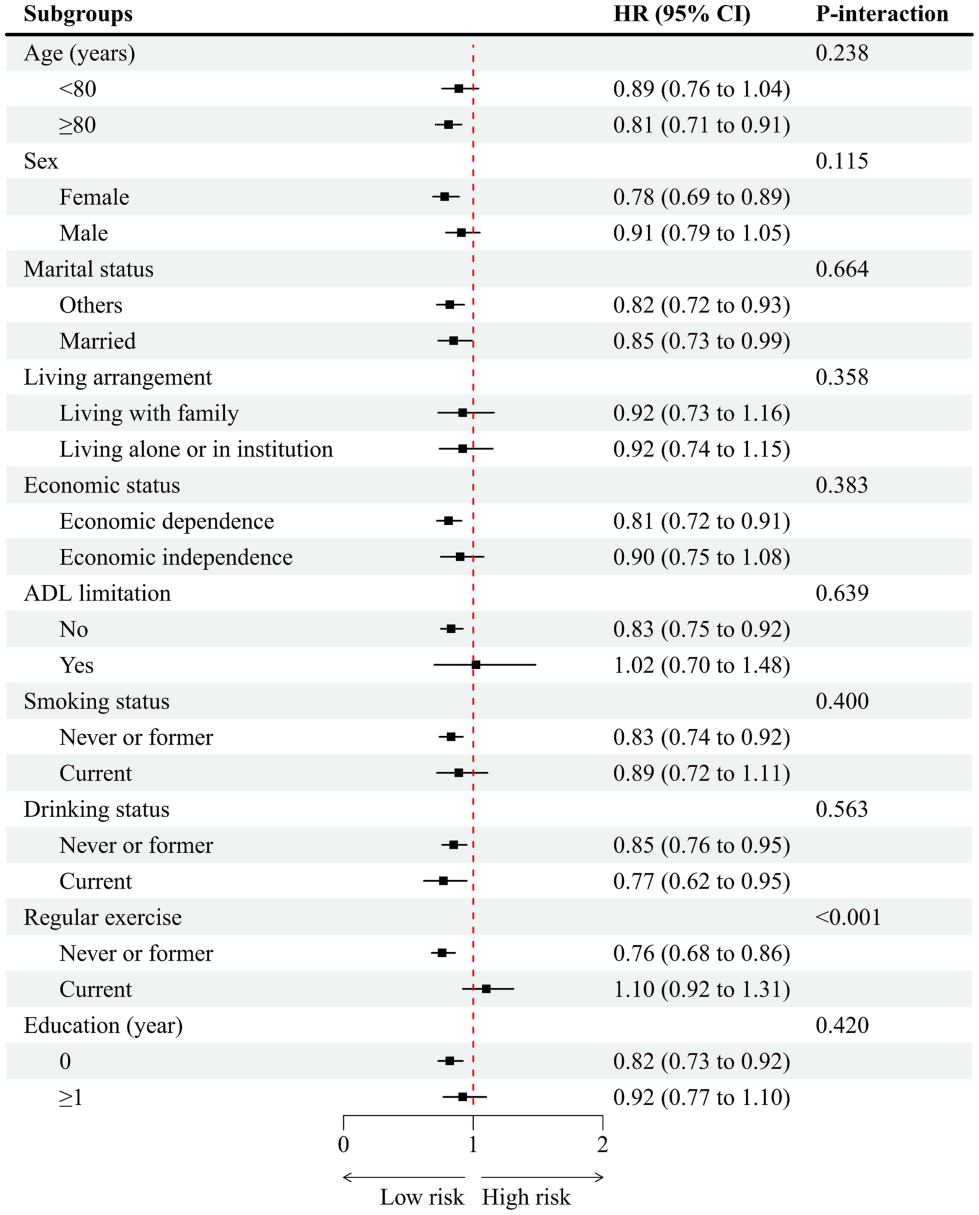
Association of plant-based diet index with hypertension stratified by participant characteristics. HR, hazard ratio; CI, confidence interval. Each stratification controlled for all factors (baseline age, sex, marital status, education, residence, living arrangement, economic status, smoking status, drinking status, regular exercise, body mass index, activity of daily living limitation, sleep time, the number of natural teeth, and denture use, heart disease, cerebrovascular disease, diabetes mellitus, respiratory disease, and cancer) except the stratification factor itself.

Subgroup analyses were performed to assess whether the protective association between high PDI and reduced hypertension risk was consistent across different population strata (Figure 3). The inverse association between higher PDI (analyzed as a continuous variable) and lower hypertension risk was generally consistent across most subgroups. Specifically, the protective effect was not significantly modified by age (<80 vs. ≥80 years), sex (male vs. female), marital status (married vs. others), living arrangement (living with family vs. living alone), economic status (independent vs. dependent), ADL limitations (yes vs. no), smoking status (never/former vs. current), drinking status (never/former vs. current), or education level (0 years vs. ≥1 year), as all corresponding *p*-values for interaction (*p*-interaction) were greater than 0.05. However, a significant interaction was observed for regular exercise (*p*-interaction < 0.001). Among participants who never or formerly exercised, higher PDI was associated with a significantly reduced risk of hypertension (HR = 0.76, 95% CI: 0.68–0.86), whereas no significant association was observed among current exercisers (HR = 1.10, 95% CI: 0.92–1.31).

### Sensitivity analyses

To assess the robustness of our findings, we conducted multiple sensitivity analyses (Supplementary Table 4). First, we performed PSM, which produced results consistent with the primary analysis. Second, in a fully adjusted model restricted to participants with complete data (*n* = 3,932), the association remained unchanged. Third, after excluding individuals with pre-existing comorbidities (including cardiovascular disease, diabetes mellitus, cerebrovascular disease, chronic respiratory disease, or cancer), the results were unaltered.

## Discussion

This cohort study investigated the association between PDI and the incidence of hypertension among older adults in China. Our findings indicate that a higher PDI is associated with a reduced risk of hypertension, with a clear dose–response relationship observed across quartiles of PDI. These findings contribute to the growing body of evidence supporting the cardiovascular benefits of plant-based diets, particularly in older adults-a demographic that has been underrepresented in prior research despite their heightened vulnerability to hypertension and related complications.

Our results align with previous studies conducted in middle-aged and mixed-age populations that suggest plant-based dietary patterns are associated with a lower risk of hypertension (2–4, 9, 10) (Supplementary Table 5). However, this study makes several unique contributions. First, we specifically focused on an older adult population (mean age >82 years), a group with distinct physiological changes, polypharmacy, and altered nutrient absorption, which may modify the diet-disease relationship. While a previous study in Chinese adults included participants aged ≥18 (11), our findings confirm that the protective association of a plant-based diet persists even in the oldest-old, extending the generalizability of this dietary strategy to advanced ages. Second, unlike studies that dichotomize diets as vegetarian or non-vegetarian (3, 4), we utilized a continuous PDI. This approach captures a spectrum of dietary habits common in China, where diets are often plant-predominant but not strictly vegetarian, thus providing more practical and culturally relevant insights. This is crucial as a recent meta-analysis found no significant effect of strict vegan diets on blood pressure in some analyses (5), suggesting that a predominance of plant foods, rather than absolute exclusion of animal products, may be the key factor. Moreover, emerging evidence indicates that not all plant-based diets are equally beneficial. An unhealthy plant-based diet high in refined grains, sugars, and processed plant foods has been positively associated with hypertension and metabolic syndrome (11). Our J-shaped dose–response curve further refines this understanding, indicating an optimal range of plant-based intake for maximum benefit.

The consistency of the inverse association between PDI and hypertension risk across various subgroups-including those stratified by economic status and ADL limitations-enhances the robustness of our findings. The lack of effect modification by economic status suggests that the benefits of a plant-based diet, often centered on affordable staple foods like vegetables, legumes, and grains in this Chinese cohort, may be accessible across socioeconomic strata. Furthermore, the persistent association among participants with ADL limitations indicates that the protective effect may hold even for individuals facing potential barriers to food preparation, supporting the broader applicability of plant-based dietary recommendations in this vulnerable age group. However, subgroup analyses revealed a significant interaction between regular exercise and the association of PDI with hypertension risk. Specifically, the protective effect of a higher plant-based diet index was more pronounced among participants who reported never or formerly engaging in regular exercise, whereas no significant association was observed among those who reported currently engaging in regular exercise. This suggests that the benefits of a plant-based diet on hypertension risk may be particularly relevant for older adults with sedentary or less active lifestyles. It is possible that physical activity itself exerts a strong protective effect against hypertension, thereby attenuating the additional benefit of diet in more active individuals. Alternatively, those who exercise regularly may already exhibit healthier overall lifestyles, reducing the marginal benefit of dietary modification. These findings highlight the potential synergistic or substitutive roles of diet and physical activity in hypertension prevention and suggest that dietary interventions may be especially beneficial in populations with limited physical activity.

Potential biological mechanisms underlie the inverse relationship between plant-based diets and hypertension risk in older adults. These diets provide key minerals-potassium, magnesium, and calcium-that regulate blood pressure by modulating sodium-potassium pump activity, relaxing vascular smooth muscle, and enhancing endothelial function (9, 12). Potassium, in particular, antagonizes sodium-induced hypertension while promoting renal sodium excretion. Plant-based diets are also rich in polyphenols (e.g., quercetin, anthocyanins), which attenuate oxidative stress-mediated vascular damage through antioxidant activity (9). Dietary nitrates from leafy greens and beets further support cardiovascular health by increasing nitric oxide bioavailability, improving endothelial function (13). Additionally, high fiber intake fosters beneficial gut microbiota (e.g., Bifidobacterium, Lactobacillus), which generate short-chain fatty acids such as propionate and butyrate. These metabolites mitigate inflammation via G protein-coupled receptor signaling and modulate the renin-angiotensin system (14, 15). Unlike saturated fats from animal sources, plant-derived unsaturated fatty acids (e.g., linolenic acid, oleic acid) enhance vascular reactivity by reducing cellular cholesterol and improving membrane fluidity. Furthermore, ω-3 fatty acids from olive oil and nuts suppress pro-inflammatory prostaglandin synthesis, contributing to improved vascular health (16).

The findings from this cohort study hold meaningful implications for clinical practice and public health strategies aimed at healthy aging. They suggest that encouraging a shift toward a diet richer in fruits, vegetables, legumes, nuts, and whole grains could be a valuable non-pharmacological strategy to mitigate hypertension incidence in older adults. For clinicians and nutritionists, this supports the promotion of dietary patterns that increase the PDI score, which does not necessitate complete elimination of animal products but rather a prioritization of plant-based sources. This flexible approach may be more acceptable and sustainable for older populations. Integrating such dietary advice into geriatric care and community health programs could contribute to reducing the substantial burden of hypertension and its associated cardiovascular complications in our aging global population. Future research should build upon these findings in several key directions. Longitudinal studies with longer follow-up periods are needed to confirm the sustained benefits of plant-based diets and to establish causal relationships. Interventional trials are warranted to test the efficacy of culturally tailored, plant-predominant dietary patterns in preventing and managing hypertension among older adults. Further investigation is also needed to elucidate the specific components of plant-based diets (e.g., polyphenols, specific fibers, or fatty acids) that are most protective, and to understand the role of the gut microbiome as a potential mediator. Finally, research should explore the effectiveness of dietary interventions in diverse ethnic and socioeconomic groups to ensure the equitable application of these findings.

## Strengths and limitations

Our study has several strengths, including its prospective cohort design, large sample size, and comprehensive adjustment for potential confounders. The use of a validated PDI allowed for a nuanced assessment of dietary patterns beyond simple vegetarian/non-vegetarian classifications. Additionally, the consistency of our findings across various subgroups enhances the robustness of our conclusions.

However, several limitations should be acknowledged. First, dietary intake was assessed using a simplified FFQ, which, while practical for large-scale epidemiological studies like the CLHLS, is inherently susceptible to measurement error. This includes recall bias, where older adults may inaccurately remember or report their habitual food intake, and social desirability bias, potentially leading to over-reporting of ‘healthy’ plant-based foods. Furthermore, the simplified nature of the FFQ, with broad frequency categories and a limited food list, may lead to the misclassification of participants’ true dietary patterns. We acknowledge that this non-differential misclassification would most likely bias our results toward the null, attenuating the observed hazard ratios. Therefore, the true protective association between a healthful plant-based diet and hypertension risk in this population may be stronger than what we reported. Second, our follow-up period (mean 3.0 years) was relatively short. While this was sufficient to identify a significant number of incident hypertension cases and a clear association with plant-based diet intake, it may constrain our ability to detect the full long-term trajectory of risk. Hypertension is a chronic condition that develops over decades, and a longer observation period would be necessary to determine if the protective association we observed is sustained, attenuates, or strengthens over time. Furthermore, a longer follow-up could provide more power to investigate how changes in dietary patterns influence hypertension risk later in life. Future studies with extended follow-up durations are warranted to confirm and build upon our findings. Third, while we adjusted for multiple confounders, residual confounding from unmeasured factors cannot be ruled out. We lacked data on medication use (e.g., non-steroidal anti-inflammatory drugs or other medications that can influence blood pressure), precise sodium/salt intake, and genetic predisposition to hypertension. If individuals with a higher PDI were also systematically more likely to avoid certain medications, consume less sodium, or have a lower genetic risk, the observed protective association could be overestimated. Conversely, if health-conscious individuals adopting plant-based diets were also more likely to be prescribed antihypertensive medications preemptively (which would lower their measured blood pressure), this could potentially lead to an underestimation of the true protective effect. Finally, while our findings provide evidence for the benefits of plant-based diets in a Chinese aging population, their generalizability to other ethnic and cultural contexts may be limited due to differences in dietary patterns and lifestyle factors. Future studies are needed to validate these associations in diverse populations and to develop culturally tailored dietary interventions for hypertension prevention globally.

## Conclusion

Our study provides evidence that higher plant-based food intake is associated with a reduced risk of hypertension in older Chinese adults. These findings align with broader literature on plant-based diets and cardiovascular health while addressing a critical gap in research on aging populations. Future interventions should consider culturally tailored dietary strategies to mitigate hypertension risk in older adults.

## Data Availability

The datasets presented in this study can be found in online repositories. The names of the repository/repositories and accession number(s) can be found in the article/Supplementary material.

## References

[1] Mills KT Stefanescu A He J. The global epidemiology of hypertension. Nat Rev Nephrol. (2020) 16:223–37. doi: 10.1038/s41581-019-0244-2, PMID: 32024986 PMC7998524

[2] da Silva FMO Pimenta AM Juvanhol LL Hermsdorff HHM Bressan J. Healthful plant-based diet and incidence of hypertension in Brazilian adults: a six-year follow-up of the CUME study. Nutrition. (2025) 133:112711. doi: 10.1016/j.nut.2025.112711, PMID: 40048766

[3] Chuang S-Y Chiu THT Lee C-Y Liu T-T Tsao CK Hsiung CA. Vegetarian diet reduces the risk of hypertension independent of abdominal obesity and inflammation: a prospective study. J Hypertens. (2016) 34:2164–71. doi: 10.1097/HJH.0000000000001068, PMID: 27512965

[4] Lee KW Loh HC Ching SM Devaraj NK Hoo FK. Effects of vegetarian diets on blood pressure lowering: a systematic review with meta-analysis and trial sequential analysis. Nutrients. (2020) 12:1604. doi: 10.3390/nu12061604, PMID: 32486102 PMC7352826

[5] Xia X Zhang J Wang X Xiong K Pan Z Wang J. Effects of vegetarian diets on blood lipids, blood glucose, and blood pressure: a systematic review and meta-analysis. Food Funct. (2024) 15:11834–46. doi: 10.1039/d4fo03449j, PMID: 39526314

[6] Lv X Li W Ma Y Chen H Zeng Y Yu X. Cognitive decline and mortality among community-dwelling Chinese older people. BMC Med. (2019) 17:63. doi: 10.1186/s12916-019-1295-8, PMID: 30871536 PMC6419492

[7] Qin A Chen C Bao B Xin T Xu L. Estimating the impact of different types hearing loss on cognitive decline and the joint effect of hearing loss and depression on cognitive decline among older adults in China. J Affect Disord. (2024) 351:58–65. doi: 10.1016/j.jad.2024.01.203, PMID: 38286235

[8] WangJTaylorAWZhangTAppletonSShiZ. Association between body mass index and all-cause mortality among oldest old Chinese. J Nutr Health Aging. (2018) 22:262–8. doi: 10.1007/s12603-017-0907-2, PMID: 29380854

[9] Tomé-Carneiro J Visioli F. Plant-based diets reduce blood pressure: a systematic review of recent evidence. Curr Hypertens Rep. (2023) 25:127–50. doi: 10.1007/s11906-023-01243-7, PMID: 37178356 PMC10224875

[10] Mokhtari E Rouhani P Shahdadian F Mohammadi S Heidari Z Saneei P. An unhealthy plant-based diet increases risk of hypertension but not Framingham risk score in adults. Curr Dev Nutr. (2023) 7:102008. doi: 10.1016/j.cdnut.2023.102008, PMID: 37869525 PMC10587703

[11] Zhao Y Gao Q Zhang J Wang J Araki T Zhao J. Trajectories of plant-based diet indices and the associated risk of hypertension among Chinese adults: a cohort study based on the China health and nutrition survey 2004-2015. Nutr J. (2024) 23:eng. 4.4. doi: 10.1186/s12937-024-01053-w, PMID: 39627809 PMC11613601

[12] Marques FZ Mackay CR Kaye DM. Beyond gut feelings: how the gut microbiota regulates blood pressure. Nat Rev Cardiol. (2018) 15:20–32. doi: 10.1038/nrcardio.2017.120, PMID: 28836619

[13] Joshi S Ettinger L Liebman SE. Plant-based diets and hypertension. Am J Lifestyle Med. (2020) 14:397–405. doi: 10.1177/1559827619875411, PMID: 33281520 PMC7692016

[14] Satija A Hu FB. Plant-based diets and cardiovascular health. Trends Cardiovasc Med. (2018) 28:437–41. doi: 10.1016/j.tcm.2018.02.004, PMID: 29496410 PMC6089671

[15] Del Re A Aspry K. Update on plant-based diets and cardiometabolic risk. Curr Atheroscler Rep. (2022) 24:173–83. doi: 10.1007/s11883-022-00981-4, PMID: 35332441

[16] Manolis AA Manolis TA Melita H Manolis AS. Features of a balanced healthy diet with cardiovascular and other benefits. Curr Vasc Pharmacol. (2023) 21:163–84. doi: 10.2174/1570161121666230327135916, PMID: 36974413

