# Supplementary material for: Association of plant-based food intake in daily diets and hypertension in older adults: a cohort study
Source: Front Public Health. 2025 Oct 28;13:1651399. doi: 10.3389/fpubh.2025.1651399 (PMC12602512; doi:10.3389/fpubh.2025.1651399)
Supplement: Supplementary file 1 [file Supplementary_file_1.docx]

**Supplementary Material**

**Supplementary Table 1** **Plant-based diet index scoring**

**Supplementary Table 2 The numbers (percentage) of the missing variables.**

**Supplementary Table 3 Demographic and clinical characteristics of the study population after propensity score matching**

**Supplementary Table 4 Sensitivity analyses for the association of PDI with hypertension.**

**Supplementary Table 5 Summary of Selected Studies on Plant-Based Diets and Hypertension.**

**Supplementary Figure 1 Flowchart of the included study population.**

**Supplementary Table 1** Plant-based diet index scoring

| **Food category** | **Food** | **Frequency** | Plant-based diet index scoring |
| --- | --- | --- | --- |
|  | Vegetable oil | Yes | 5 |
|  |  | No | 1 |
|  | Fresh fruit | Almost everyday | 5 |
|  |  | Quite often | 4 |
|  |  | Occasionally | 2 |
|  |  | Rarely or never | 1 |
|  | Fresh vegetable | Almost everyday | 5 |
|  |  | Quite often | 4 |
|  |  | Occasionally | 2 |
|  |  | Rarely or never | 1 |
|  | bean products | Almost everyday | 5 |
|  |  | ≥1 time/week | 4 |
|  |  | ≥1 time/month | 3 |
|  |  | Occasionally | 2 |
|  |  | Rarely or never | 1 |
|  | Garlic | Almost everyday | 5 |
|  |  | ≥1 time/week | 4 |
|  |  | ≥1 time/month | 3 |
|  |  | Occasionally | 2 |
|  |  | Rarely or never | 1 |
|  | Nut | Almost everyday | 5 |
|  |  | ≥1 time/week | 4 |
|  |  | ≥1 time/month | 3 |
|  |  | Occasionally | 2 |
|  |  | Rarely or never | 1 |
|  | Tea | Almost everyday | 5 |
|  |  | ≥1 time/week | 4 |
|  |  | ≥1 time/month | 3 |
|  |  | Occasionally | 2 |
|  |  | Rarely or never | 1 |
|  | Mushroom | Almost everyday | 5 |
|  |  | ≥1 time/week | 4 |
|  |  | ≥1 time/month | 3 |
|  |  | Occasionally | 2 |
|  |  | Rarely or never | 1 |
|  | Sugar | Almost everyday | 5 |
|  |  | ≥1 time/week | 4 |
|  |  | ≥1 time/month | 3 |
|  |  | Occasionally | 2 |
|  |  | Rarely or never | 1 |
|  | Salt-preserved vegetable | Almost everyday | 5 |
|  |  | ≥1 time/week | 4 |
|  |  | ≥1 time/month | 3 |
|  |  | Occasionally | 2 |
|  |  | Rarely or never | 1 |
| Animal food | Animal fat | Yes | 1 |
|  |  | No | 5 |
|  | Meat | Almost everyday | 1 |
|  |  | ≥1 time/week | 2 |
|  |  | ≥1 time/month | 3 |
|  |  | Occasionally | 4 |
|  |  | Rarely or never | 5 |
|  | Fish | Almost everyday | 1 |
|  |  | ≥1 time/week | 2 |
|  |  | ≥1 time/month | 3 |
|  |  | Occasionally | 4 |
|  |  | Rarely or never | 5 |
|  | Egg | Almost everyday | 1 |
|  |  | ≥1 time/month | 3 |
|  |  | Occasionally | 4 |
|  |  | Rarely or never | 5 |
|  | Dairy products | Almost everyday | 1 |
|  |  | ≥1 time/week | 2 |
|  |  | ≥1 time/month | 3 |
|  |  | Occasionally | 4 |
|  |  | Rarely or never | 5 |

**Supplementary table 2** The numbers (percentage) of the missing variables

| Characteristics ^a^ | Number (%) with missing data |
| --- | --- |
| Economic status | 0.30 |
| Sleep time | 0.28 |
| Natural tooth number | 0.38 |
| Body mass index | 0.45 |
| cancer | 0.10 |

^a^ List only the variables with missing data.

**Supplementary Table 3** Demographic and clinical characteristics of the study population after propensity score matching

| Characteristics | Total (n = 3212) | Plant-based diet index | | P value |
| --- | --- | --- | --- | --- |
|  |  | Low level (n = 1606) | High level (n = 1606) |  |
| Age (year), mean (SD) | 83.16 (11.03) | 83.12 (11.10) | 83.20 (10.96) | 0.845 |
| Female, no. (%) | 1706 (53.1) | 850 (52.9) | 856 (53.3) | 0.860 |
| Residence, no. (%) |  |  |  | 0.399 |
| Urban area | 452 (14.1) | 215 (13.4) | 237 (14.8) |  |
| Town area | 698 (21.7) | 343 (21.4) | 355 (22.1) |  |
| Rural area | 2062 (64.2) | 1048 (65.3) | 1014 (63.1) |  |
| Married, no. (%) | 1346 (41.9) | 680 (42.3) | 666 (41.5) | 0.642 |
| Living arrangement, no. (%) |  |  |  | 0.674 |
| living alone | 552 (17.2) | 281 (17.5) | 271 (16.9) |  |
| Living with family | 2660 (82.8) | 1325 (82.5) | 1335 (83.1) |  |
| Smoking status, no. (%) |  |  |  | 0.876 |
| Never | 2051 (63.9) | 1019 (63.4) | 1032 (64.3) | 0.876 |
| Current | 695 (21.6) | 353 (22.0) | 342 (21.3) |  |
| Former | 466 (14.5) | 234 (14.6) | 232 (14.4) |  |
| Drinking status, no. (%) |  |  |  | 0.865 |
| Never | 2121 (66.0) | 1056 (65.8) | 1065 (66.3) |  |
| Current | 693 (21.6) | 346 (21.5) | 347 (21.6) |  |
| Former | 398 (12.4) | 204 (12.7) | 194 (12.1) |  |
| Regular exercise, no. (%) |  |  |  | 0.664 |
| Never | 1871 (58.3) | 948 (59.0) | 923 (57.5) |  |
| Current | 1007 (31.4) | 493 (30.7) | 514 (32.0) |  |
| Former | 334 (10.4) | 165 (10.3) | 169 (10.5) |  |
| Education (year), no. (%) |  |  |  | 0.734 |
| 0 | 1850 (57.6) | 924 (57.5) | 926 (57.7) |  |
| 1-6 | 1024 (31.9) | 519 (32.3) | 505 (31.4) |  |
| >6 | 338 (10.5) | 163 (10.1) | 175 (10.9) |  |
| Economic independence, no. (%) | 552 (17.2) | 281 (17.5) | 271 (16.9) | 0.674 |
| Number of natural teeth, no. (%) |  |  |  | 0.882 |
| <10 | 1935 (60.2) | 971 (60.5) | 964 (60.0) |  |
| 10-20 | 597 (18.6) | 293 (18.2) | 304 (18.9) |  |
| ≥20 | 680 (21.2) | 342 (21.3) | 338 (21.0) |  |
| Denture use, no. (%) | 950 (29.6) | 482 (30.0) | 468 (29.1) | 0.615 |
| BMI (kg/m^2^), no. (%) |  |  |  | 0.762 |
| Underweight (<18.5) | 1812 (56.4) | 915 (57.0) | 897 (55.9) |  |
| Normal (18.5-24) | 1032 (32.1) | 515 (32.1) | 517 (32.2) |  |
| Overweight (24-28) | 299 (9.3) | 141 (8.8) | 158 (9.8) |  |
| Obese (≥28) | 69 (2.1) | 35 (2.2) | 34 (2.1) |  |
| ADL limitation, no. (%) | 282 (8.8) | 137 (8.5) | 145 (9.0) | 0.663 |
| Diabetes mellitus, no. (%) | 50 (1.6) | 23 (1.4) | 27 (1.7) | 0.669 |
| Heart disease, no. (%) | 163 (5.1) | 79 (4.9) | 84 (5.2) | 0.748 |
| Cerebrovascular disease, no. (%) | 105 (3.3) | 53 (3.3) | 52 (3.2) | 1.000 |
| Respiratory disease, no. (%) | 318 (9.9) | 161 (10.0) | 157 (9.8) | 0.859 |
| Cancer, no. (%) | 8 (0.2) | 3 (0.2) | 5 (0.3) | 0.723 |

*BMI* body mass index, *ADL* activity of daily living.

Values are presented as number (%) or mean ± SD. Differences in characteristics were compared using the χ^2^ test for categorical variables and t-test for continuous variables.

**Supplementary Table 4** Sensitivity analyses for the association of plant-based diet index (PDI) with hypertension.

| PDI Category | Removing participants with missing covariate data | Removing participants who had heart disease, diabetes mellitus, cerebrovascular disease, respiratory disease, or cancer | After propensity score matching |
| --- | --- | --- | --- |
|  | HR (95% CI) | HR (95% CI) | HR (95% CI) |
| By median | | | |
| Low level (<48) | Reference | Reference | Reference |
| High level (≥48) | 0.85 (0.77-0.93) | 0.83 (0.74-0.92) | 0.83 (0.75-0.92) |
| By Quartile | | | |
| Q1 (Lowest, ≤43) | Reference | Reference | Reference |
| Q2 (43–48) | 0.87 (0.76-0.99) | 0.84 (0.72-0.96) | 0.89 (0.77-1.02) |
| Q3 (48–53) | 0.81 (0.71-0.93) | 0.77 (0.66-0.89) | 0.80 (0.69-0.93) |
| Q4 (Highest, >53) | 0.79 (0.68-0.90) | 0.80 (0.69-0.93) | 0.81 (0.69-0.94) |
| P for trend | <0.001 | 0.002 | 0.002 |
| PDI score | 0.99 (0.98-0.99) | 0.99 (0.98-0.99) | 0.99 (0.98-0.99) |

*HR* hazard ratio, *CI* confidence interval.

Notes: Multivariate models were adjusted for baseline age, sex, marital status, education, residence, living arrangement, economic status, smoking status, drinking status, regular exercise, body mass index, activity of daily living limitation, sleep time, the number of natural teeth, and denture use, heart disease, cerebrovascular disease, diabetes mellitus, respiratory disease, and cancer.

**Supplementary Table 5** Summary of Selected Studies on Plant-Based Diets and Hypertension.

| **Study (Year, Country)** | **Study Design** | **Population Characteristics** | **Dietary Assessment Method** | **Main Findings Related to**  **Plant-Based Diets and**  **Hypertension** |
| --- | --- | --- | --- | --- |
| da Silva et al. (2025), Brazil [2] | Prospective Cohort | Brazilian adults (mean age ~34 yrs) | Food frequency questionnaire (FFQ); healthy plant-based diet index (hPDI) | Higher hPDI was associated with a lower incidence of hypertension over 6 years. |
| Chuang et al. (2016), Taiwan [3] | Prospective Cohort | Taiwanese Buddhists (≥20 yrs) | FFQ; vegetarian status | Vegetarians had a 34% lower risk of hypertension than non-vegetarians, independent of obesity and inflammation. |
| Lee et al. (2020), Korea [4] | Systematic Review & Meta-analysis | Mixed-age populations (RCTs) | Various | Vegetarian diets are associated with significant reductions in BP compared with omnivorous diets |
| Xia et al. (2024), Meta-analysis [5] | Systematic Review & Meta-analysis | Mixed-age populations (Observational & RCTs) | Various | Found no significant association between vegetarian diets and blood pressure in the analysis pooling observational and intervention studies. |
| Tomé-Carneiro et al. (2023), Systematic Review [9] | Systematic Review | Mixed-age populations (Observational & RCTs) | Various | Plant-based diets are associated with lower blood pressure and overall better health outcomes (namely, on the cardiovascular system) when compared to animal-based diets |
| Mokhtari et al. (2023), Iran [10] | Cross-sectional | Middle-aged adults | FFQ; PDI | An unhealthy plant-based diet (high in refined grains, sugars) was associated with a higher risk of hypertension. |
| Zhao et al. (2024), China [11] | Prospective Cohort | Chinese adults (≥18 yrs) from CHNS | 24-hour dietary recalls; trajectory of PDI | Favorable trajectories of PDI (increasing healthy plant-based intake) were associated with a lower risk of hypertension. |
| Joshi et al. (2020), Meta-analysis [13] | Systematic Review & Meta-analysis | Mixed-age populations from intervention trials | Various (Intervention studies) | Plant-based dietary interventions significantly reduced systolic and diastolic blood pressure. |

*PDI* Plant-Based Diet Index, *hPDI* healthy Plant-Based Diet Index, *FFQ* Food Frequency Questionnaire, *CHNS* China Health and Nutrition Survey, *RCT* Randomized Controlled Trial.


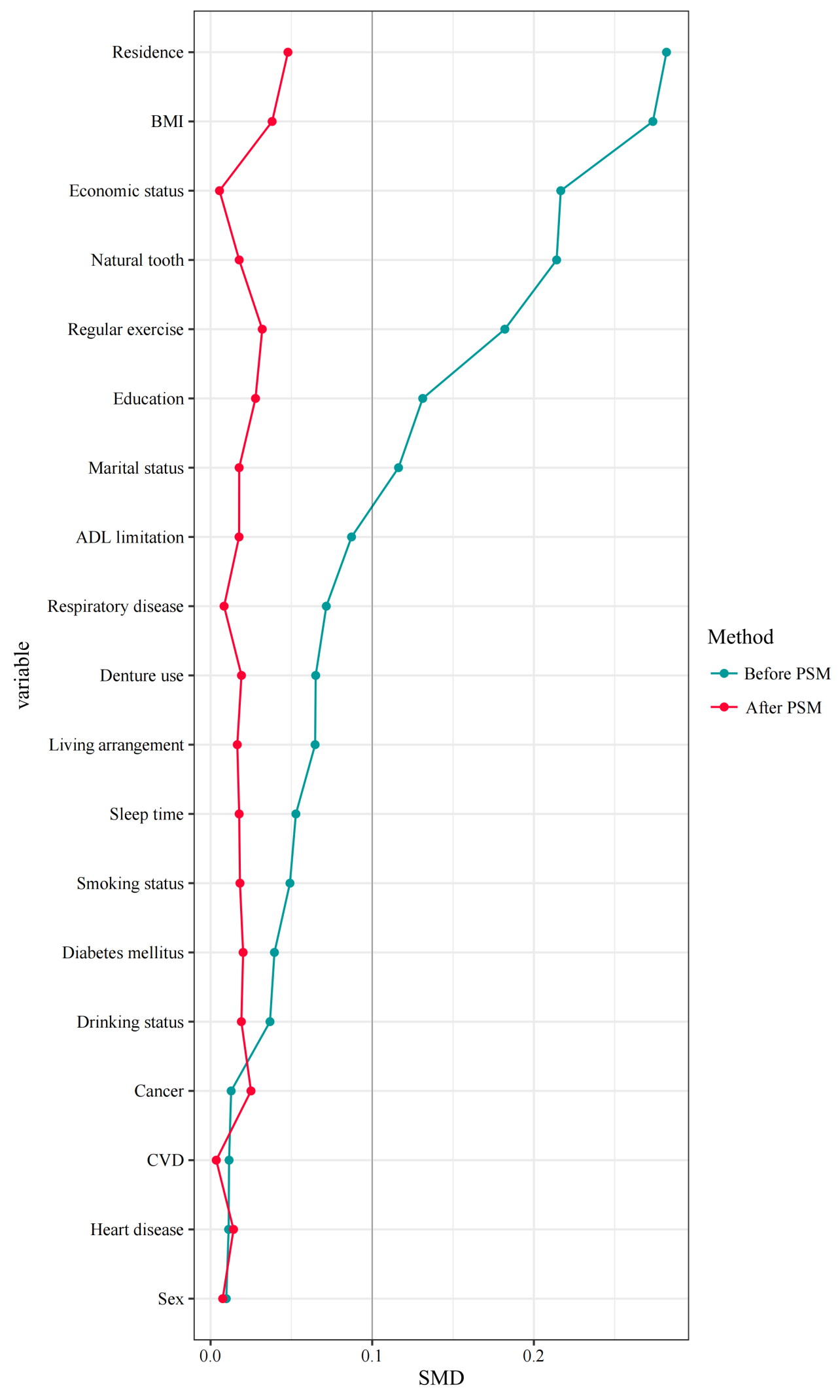


**Supplementary Figure 1** The standardized mean differences (SMD) of the variables.

*BMI* body mass index, *ADL* activities of daily living, *CVD* cerebrovascular disease, *PSM* propensity score matching.
